# Patients’ Perspectives of Factors That Influence Pharmacogenetic Testing Uptake: Enhancing Patient Counseling and Results Dissemination

**DOI:** 10.3390/jpm12122046

**Published:** 2022-12-11

**Authors:** Diliara Bagautdinova, Christelle Lteif, Elizabeth Eddy, Joshua Terrell, Carla L. Fisher, Julio D. Duarte

**Affiliations:** 1 Department of Advertising, College of Journalism and Communications, University of Florida, Gainesville, FL 32611, USA; 2 Center for Pharmacogenomics and Precision Medicine, College of Pharmacy, University of Florida, Gainesville, FL 32610, USA; 3 Department of Pharmacotherapy and Translational Research, College of Pharmacy, University of Florida, Gainesville, FL 32610, USA; 4 Department of Health Outcomes and Biomedical Informatics, College of Medicine, University of Florida, Gainesville, FL 32610, USA

**Keywords:** pharmacogenetic testing, medically underserved population, patient preferences, qualitative research

## Abstract

Patient preferences for pharmacogenetic (PGx) counseling, testing and results dissemination are not well-established, especially in medically underserved Black and Latino populations. The aim of this study was to capture the preferences of Black and Latino patients who received PGx testing to ascertain: (1) factors enhancing their willingness to do testing and (2) preferences for the dissemination of results. Using the constant comparative method, we thematically analyzed interviews with 13 patients from medically underserved populations who had undergone PGx testing. The findings describe participants *wanting better medication options, receiving a clear explanation about the testing, valuing or having an interest in science or medicine* and *having misconceptions about testing results* as factors affecting one’s willingness to undergo PGx testing. Additionally, patients confirmed preferring *receiving results of PGx testing in a sharable format* and described the significance of *discussing results in a clinical appointment*. The findings provide insight into what Black and Latino patients may prefer in terms of clinical implementation of PGx testing. These results can be utilized for tailoring future implementation of PGx testing and informing best pre- and post-test patient counseling and education practices.

## 1. Introduction

Despite substantial evidence showing that patient genetics can influence response to specific medications, implementing pharmacogenetic (PGx) testing into clinical practice remains relatively uncommon outside of the field of oncology. The slowness with which PGx testing moved to clinical practice could potentially be contributing to healthcare disparities, as we have previously shown that medically underserved patients are more often prescribed medications that have PGx guidelines available [1]. In addition, there are few reports of patient perceptions and preferences related to PGx testing, with most previous studies focusing on primarily white patient populations [2]. Thus, to speed clinical implementation of PGx testing, it is important to identify potential barriers preventing its uptake. Some barriers to implementation strategies include the amount of evidence of cost-effectiveness, the availability of support tools for PGx integration and acceptance of PGx testing among healthcare professionals and patients [3,4]. The situation among healthcare providers has been studied extensively in recent years. However, patient preferences related to PGx counseling and testing are not well-established, particularly in medically underserved populations.

To enhance clinical implementation of PGx testing, clinician-implementation scientists are working to establish best practices for patient counseling on PGx testing [5,6,7]—practices that should be informed by the stakeholders’ (especially patients’) preferences. A recent scoping review of research addressing patients’ PGx testing knowledge and experiences demonstrated the important but often missing voice of patients, particularly diverse and marginalized groups such as Black and Latino populations [8]. Synthesized findings indicated that patients have both positive and negative psychological responses to PGx testing but that they also do not always understand the testing or results. They may also perceive potential harms from undergoing testing, whereas perceived benefits (e.g., less side effects from medications) may promote uptake. The scoping review concluded that pre- and post-PGx testing counseling should attend to patients’ perceived benefits of testing, facilitate patient comprehension of testing, acknowledge emotional responses to testing and address results. A recent study of pre- and post-test counseling at four sites provided further evidence that these may be best practices in patient counseling and education [9].

The scoping review also revealed that patients want their results disseminated to them in a comprehensible manner that promotes better care, which may enhance the patient–clinician relationship. This may be especially important with underserved Black and Latino patients who report mistrust as a factor inhibiting their willingness to engage in genomic medicine [8]. Studies have yet to explore patients’ perspectives of optimal dissemination of PGx results [7] and studies on underserved patients’ perspectives are nearly non-existent [8]. There is a need for more research capturing patients’ preferences, particularly their education needs regarding testing, to establish best practice for pre- and post-test patient counseling.

Better understanding of patients’ preferences related to PGx testing should greatly increase the likelihood of patient acceptance and, therefore, implementation success. With this in mind, the aim of this study was to capture the preferences of patients from medically underserved backgrounds who underwent clinical PGx testing to identify: (1) factors that enhance their willingness to do testing and (2) preferences for the dissemination of results.

## 2. Materials and Methods

### 2.1. Participant Recruitment

Participants were recruited from a larger pilot study of PGx clinical implementation in Black and Latino patients within the University of Florida Health System (UF Health). Patients were eligible to participate in the pilot study if they were adults who self-identified as Black or Latino, experienced a medication change within the past 6 months and were prescribed a medication that could be informed by the PGx testing panel offered by UF Health. Participation in a semi-structured interview was optional. Therefore, study participants had to agree to take part in the interview portion of the study. The study was approved by the University of Florida Institutional Review Board and all patients provided written informed consent prior to their participation.

Once the PGx test was completed, the results were entered in the Electronic Health Record and Clinical Decision Support Best Practice Advisories alerted providers if a PGx interaction might occur with a medication they prescribed. A clinical note, written by a UF Health Precision Medicine Program pharmacist, provided basic guidance on the most clinically impactful results and was forwarded to the provider for review. The patients had access to the results as well as the clinical notes in their records. All clinical medication decisions were made at the discretion of the provider.

### 2.2. Data Collection

Two research coordinators trained by the lead qualitative expert [CF] conducted all in-depth, semi-structured, audio-recorded interviews by phone or via Zoom between December 2021 and April 2022. Participants were asked what motivated them to agree to complete PGx testing, what factors made them initially resistant or hesitant to undergo the testing and their perspective on what would motivate or inhibit others’ willingness to undergo PGx testing (e.g., What enhanced your willingness to complete PGx testing? What would make you unwilling to undergo preemptive PGx testing?). Participants were also asked to describe their preferences for receiving PGx testing results (e.g., When you received your PGx test results, what do you recall liking about that? What didn’t you like?). They were also asked specifically about their preferences regarding a shareable format (e.g., a results card). Interviews lasted on average 45 min and were professionally transcribed resulting in 127 single-spaced pages of data.

### 2.3. Data Analysis

The lead author [DB] led the thematic analysis of the data using a constant comparative method (CCM) approach [10,11]. Analysis was validated by a second coder, a qualitative expert [CF]. Data was managed using ATLAS.ti. Analysis included three analytical steps in line with CCM: (1) immersing oneself in the data reading all transcripts; (2) assigning codes (labels) to patterns using open coding; (3) collapsing codes into categories to identify themes; and (4) conducting axial coding (i.e., analyzing text associated with each theme) to establish thematic properties in order to better define themes [10,11]. To ensure rigor, the first and senior authors met regularly across the analysis process to develop a codebook, which was consistently refined as data was constantly added and compared with previous findings. Data collection and analysis were also concurrent to ensure thematic saturation was met using Owen’s (1984) criteria (repetition, recurrence and forcefulness) [12]. Thematic saturation was evident after 8 interviews. An additional 5 were recruited and enrolled to confirm thematic saturation while strengthening saturation at the property level (i.e., axial coding). Verification strategies were used across the study to ensure trustworthiness (i.e., rigor) including concurrent data collection and analysis to reach thematic saturation, using multiple coders, purposively sampling and ensuring methodological coherence [13]. Exemplar excerpts illustrate themes and are identified with important contextual data (e.g., race, ethnicity, sex, age) while maintaining confidentiality.

## 3. Results

### 3.1. Participants

In total, 13 participants underwent interviews. A majority of the interviewees identified as Black, had some education beyond high school and at least some comfort with healthcare terminology (Table 1). Table 2 reports on participants’ PGx panels and medications.

### 3.2. Motivating Factors

Participants described four factors that they perceived affect one’s willingness or motivation to undergo PGx testing: (1) *wanting better medication options*, (2) *receiving a clear explanation about the testing*, (3) *valuing or having an interest in science or medicine* and (4) *having misconceptions about testing results* (see Table 3). Each factor (i.e., theme) is illustrated using patients’ narrative accounts and further defined with thematic properties (italicized) to highlight both their education needs in pre- or post-test counseling and illustrate [7] how each factor is an important consideration in motivating patients to undergo PGx testing.

#### 3.2.1. Wanting Better Medication Options

Patients were motivated by their desire for better medication options. They described two related reasons. They perceived that PGx testing would help them *understand the impact of medications on their body*. They wanted to know how their bodies might tolerate or react to medications, particularly new medications and stressed the importance of knowing risks or side effects:

“I really didn’t know the reactions [of] some of the medicines that I take, … what they consist of and the reactions that my body has. … [With testing] I would know how it’s going to affect me and affect my body.”

(Black/African descent, non-Hispanic female, age 61)

Sometimes, their previous medication experiences informed their desire for this information:

“I really wanted to know how certain medicines will affect my body when I’m taking them and it feels like it would give me an extra sense of relief to know a little bit beforehand before I start medicine. … [With] most medicines I get all of the side effects.”

(Black/African descent, non-Hispanic female, age 24)

Patients also viewed PGx testing as an opportunity to *receive optimal medications for themselves*. In terms of optimization, patients described wanting different or “optimal” medications than those they were currently prescribed, which included “cheaper” medications and reducing the number of medications needed:

“I had several conditions. I take a lot of medication. And if they can find certain medicines that are optimal for me and get rid of some of the other ones, that’d be great. Because I take about seven different medications. So, if we can reduce that and narrow it down, that’d be fantastic.”

(White/European descent, Hispanic male, age 50)

#### 3.2.2. Receiving a Clear Explanation about the Testing

Patients acknowledged that they would be willing to undergo PGx testing if the clinician provided a clear explanation, which included four components. Patients perceived their willingness was enhanced if clinicians *explicate what PGx testing is.* Since they weren’t aware of the test beforehand and still had misconceptions about what it was used for, a clear explanation helped them understand it and reduce concerns. Explication included using language that was tailored to patients’ needs and considered health literacy (i.e., not using jargon):

“Not understanding is what makes it so scary. … Knowledge is power, you know? You can make your own decisions now and say, ‘Well, could we look for something else?’ … Take charge of your own treatment. … The doctor will explain to you in just a certain way, but if you don’t speak med-ish, then sometimes you’re left behind, just with this look in your face, like, ‘What did he just say?’ You need a dictionary sometimes.”

(White/European descent, Hispanic male, age 50)

Patients also wanted the clinician to *describe the benefits* of PGx testing. They wanted to know how the test could help them, which included more details (e.g., using examples) about how the testing benefited them:

“Explain [it] in detail, … how it helped others … like [use] examples on how the pharmacogenetic testing helped others and see the response to the medicines. … I feel like the only reason it would make me not want to [do the testing] is if you didn’t explain it more in detail or if it wasn’t explained in detail of how it would benefit that person.”

(Black/African descent, non-Hispanic female, age 24)

Patients also noted that clinicians should *clarify that the testing is logistically feasible*. Feasibility included clarifying that the test was not expensive, not painful, did not require travel (i.e., it was done at their clinician’s office) and did not involve more time (i.e., out of their day). Patients expressed that time was also an issue regarding receiving results:

“I would say, … how long it takes to get the results because you may need the medication sooner than the results get back and then that has a lot to do on whether or not you take the medicine. … Because if I need the medicine now and the results take a couple of weeks, then I’m just going to take the medicine and then deal with the results later.”

(Mixed race, non-Hispanic female, age 45)

Finally, patients wanted clinicians to *address privacy concerns*, which included a misunderstanding that the testing could reveal DNA and related information (e.g., that they weren’t related to their family members). Additionally, they expressed uncertainty about the safeguarding of their information or about their “personal information getting leaked out” (Black/African descent, non-Hispanic male, age 44).

#### 3.2.3. Valuing or Having an Interest in Science or Medicine

Patients were also motivated to carry out the PGx testing because they valued or had an interest in medicine or the advancement of science. This included two related issues. Patients acknowledged *wanting to contribute to science or research*. By testing, they perceived that they could contribute to the advancement of scientific knowledge:

“I’m always looking to do my part in helping … research [to] come up with a better way of doing things. And the feedback is one of the ways I could help by participating. My responses can be helpful in the long run for the research. … If I could be a help for anyone else by providing that feedback, that’s what I wanted to do.”

(Black/African descent, non-Hispanic male age 44)

Patients’ interest in testing was also informed by *having scientific or medical curiosity*. This curiosity was linked to wanting more scientific knowledge about their own care:

“My biggest reason is I’m always curious about science. So, I’m curious how my medication can help the conditions that I have. I had several conditions. I take a lot of medication.”

(White/European descent, Hispanic male, age 50)

#### 3.2.4. Having Misconceptions about Testing Results

Patients also described motivations to undergo PGx testing that reflected misconceptions about what the testing could tell them. They described two reasons that reflected misconceptions. They perceived that PGx testing was an opportunity to *identify genetic predispositions*:

“I want to know what could be a possible side effect for me compared to my genetics. … I was at the hospital. … my blood pressure was like 200 and something over 200. … And I couldn’t tell you today what made it rise that high. I don’t know. … I have to say that it could be genetics. … It’s a whelm of things that could happen and we don’t know.”

(Black/African descent, non-Hispanic female, age 62)

Related to this was that, by having information about genetic predispositions to certain illnesses, they were motivated to do the testing to *share information with family members*:

“There’s a genetic test that they want me to do now in the ophthalmologist’s office. They say that certain people with a certain genetic code are predisposed to lattice degeneration of the eye. So, if that’s the case, then maybe, you know, we can find out if my son has the same genetic code and is predisposed to the same thing and you could prevent it before his retina detaches. Mine detached and it’d be great to prevent it. … So, anything like that, you know, because I’m already at a certain age, but if this can help my son, somebody else in this capacity, then it’s worth it.”

(White/European descent, Hispanic male, age 50)

### 3.3. Preferences for Results Dissemination

We also asked patients about their preferences for receiving PGx testing results to inform the development of a future intervention. This included asking about a shareable format. In addition to confirming they would prefer this dissemination *preference (receiving results in a sharable format),* they also described the importance *of discussing results in a clinical appointment* (see Table 4). Their preferences for each approach (i.e., themes) are further described below with thematic properties that explicate their reasoning.

#### 3.3.1. Having Results in a Sharable Format

When prompted, all patients reported wanting a shareable format delivered to them either electronically (e.g., in email or their patient portal where it could then be stored) or in hard paper copy (e.g., via mail). They described four reasons why a shareable format was needed. Patients perceived that a sharable format could *promote better care management across providers*. Patients perceived it would be easier for them to share results with other clinicians. They perceived the results as additional information central to promoting better care.

“If [the results] helps them diagnose me quicker or more effectively, because they have a better understanding of what’s in my genetic makeup. … I would think that they would relay it through the notes that they send to the other doctor, so that they would be apprised of it before I go, so that I don’t have to explain a lot of stuff.”

(Mixed race, non-Hispanic female, age 45)

Patients also liked a shareable format so they could *share results with family members*. This was important both for their own future care (i.e., family members would have their health information) and because they believed family members could benefit from that information themselves:

“They need to know because [it] could be them in [the] same situation one day. … I would explain it definitely to my family. I would show them the graph and everything too, so that they could know if you go to the doctor, something’s going on. You could say, ‘Well, my mother had this genetic makeup probably from her parents.’”

(Black/African descent, non-Hispanic female, age 62)

Patients also described how a sharable format gave them an opportunity to *understand and digest information on their own*. They could spend more time with the information and revisit it:

“I would’ve liked the results to have come to me. … Granted, I don’t have MD behind my name, but I do have enough wherewithal and knowledge and degrees to understand stuff that I read or to go research it if it’s something I don’t know. … [Sharing] with the portal is another way that you can give more in-depth detail, where the person can read it. … I can read it then I can always come back to it.”

(Mixed race, non-Hispanic female, age 45)

Finally, patients thought that a sharable format could *provide them with more detailed results*. As the following patient shared, they also expressed that if they received it by mail more details could be included:

“The more specific and the more, not simple, but the more detail they put in it, the more it is. Because like I said, I can get lost really easy. … And the bigger the words that they use, the harder it is for me.”

(White/European descent, Hispanic female, age 48)

#### 3.3.2. Having a Clinical Appointment

Patients wanted to receive results during an in-person, telemedicine, or phone clinical appointment. Although the majority voiced a preference for an in-person appointment, those preferring telemedicine wanted to reduce the risk of COVID-19 or reduce burden in terms of travel and time. Collectively, a clinical appointment was desired for three reasons. Patients wanted the clinician to *explain the meaning of results*. They wanted the clinician’s verbal explanation so that they understood what the results meant:

“I just want him to break it down easily so I can understand. … He’s going to be more informational than a pamphlet and he’s going to break it down. And I want him to just take his time and break it down to me.”

(Black/African descent, non-Hispanic female aged 51)

Patients also wanted to *discuss the relevance of results to current medications*. They wanted to know how the results could ultimately improve their health by influencing their current care regimen:

“She [clinician] went in detail and explained it [PGx results] to me. … One of the main things was just knowing certain medications that we were talking about then that were actually on it and it was describing how it would affect me. So, I liked that it was already some medicines that I was taking that I was able to know if it was good for my body at that moment.”

(Black/African descent, non-Hispanic female, age 24)

Patients also wanted *to ask questions about their PGx testing results*. By having a clinical interaction, they could receive answers in the moment rather than waiting for answers:

“That way [having an appointment] if something was said that I didn’t understand, I can get clarity, versus someone sending me an electronic communication that just tells me, ‘Here’s your results.’ But if it’s something on there that I didn’t understand, I would have to reply to it and then wait for someone to get back to me. And the unknown is nerve-racking.”

(Black/African descent, non-Hispanic male, age 44)

## 4. Discussion

The current study validates previous research on patients’ perspectives of PGx testing [7,9], while prioritizing the marginalized voices of Black and Latino patients. To our knowledge, this is the first study to exclusively interview Black and Latino patients—two traditionally underserved populations [7,8]. Several findings parallel those identified in previous studies of other patients’ knowledge and experiences with PGx testing [7].

Patients described being motivated to do PGx testing when they perceived benefits, including improved medication selection or dosing, the implication of the results for family members, or even contributing to scientific knowledge [7]. They also described factors that might inhibit testing uptake, such as privacy concerns or not receiving clear explanations, highlighting the need for clinicians to communicate in ways that promote comprehension and ensure adequate education. Patients also shared misconceptions regarding the implications of the results, which seem to be tied to the perception that PGx testing can predict features beyond drug response, such as disease susceptibility. Collectively, these findings support the notion that medically underserved patient populations share similar perceptions and preferences related to PGx testing as other non-marginalized patient populations. These findings also offer further evidence that addressing these issues are best practices in pre-test patient counseling to promote implementation of PGx testing in clinical settings [7].

The findings also extend our understanding of a critical education need during patients’ pre- and post-test counseling that could further promote PGx testing uptake. Patients not only want their results disseminated, but they have preferences that could inform best practices for dissemination. Patients want to discuss their PGx results with their healthcare provider. They expressed the importance of having these interactions so that they had the opportunity to ask questions about what the results mean for their medication regimen. They expressed a preference for receiving their results during a clinical appointment, corresponding with their desire for their provider to interpret the results and answer any questions they may have. Such clinical interactions will also promote trust and enhance the provider–patient relationship [7,14]. This explanation of test results is also an important factor affecting how patients perceive the value of testing. Furthermore, when we probed deeper regarding preferences for results dissemination, we found patients would ideally prefer their results conveyed in a shareable method to facilitate dissemination to other healthcare providers that they might receive care from.

## 5. Limitations

While our focus on underserved patient populations is a major strength, our study also has limitations. First, patients received PGx testing for free, so cost was not extensively discussed in our interviews. Second, our sample size was relatively small with a majority of women compared to other studies. Given that we reached thematic saturation, we do not expect our results would differ if we had interviewed additional patients. However, a larger sample would further validate findings and potentially extend the results. Third, while the findings validate and extend other studies [7,9] that included multiple sites or synthesized findings found across studies, these participants represent a population within one site. Multiple sites would be optimal in future studies.

## 6. Conclusions

In summary, our findings provide insight into what Black and Latino patients may prefer in terms of clinical implementation of PGx testing. These results can be used to tailor future PGx testing implementation and inform best practices in pre- and post-test patient counseling and education.

## Figures and Tables

**Table 1:** Patient Demographics and Socioeconomic Measures.

Characteristic	N = 13
Age (years)	45.1 (±13.0)
Female	10 (76.9)
Race	
Black/African Descent	8 (61.5)
White/European Descent	4 (30.8)
Other or Mixed Descent	1 (7.7) *
Hispanic/Latino	4 (30.8)
Employment	
Full time	4 (30.8)
Disabled	5 (38.5)
Retired	1 (7.7)
Homemaker Student	1 (7.7) 1 (7.7)
Health Literacy	
Extremely Uncomfortable	0 (0)
Very Uncomfortable	0 (0)
Somewhat Uncomfortable	0 (0)
Neutral	4 (30.8)
Somewhat Comfortable	3 (23.1)
Very Comfortable	5 (38.5)
Extremely Comfortable	1 (7.7)
Income	
<$25,000	5 (38.5)
$25,001–50,000	2 (15.4)
$50,001–$75,000	0 (0.0)
$75,001–100,000	0 (0.0)
$>100,000	1 (7.7)
Don’t know/refused to answer	5 (38.5)
Education	
Some high school	1 (7.7)
High school/GED	2 (15.4)
Some college, specialized training, or technical school	8 (61.5)
Master’s Degree	1 (7.7)
Doctoral degree	1 (7.7)
Values are expressed as mean (±SD) or N (%)	

* Participant of Asian/White/American Indian/Black descent.

**Table 2:** Genes/Variants Tested with the PGx Panel and Patients’ Results.

Gene/Genotype	N = 13
*CYP2C19*^†^ (tested for *2, *3, *4, *6, *8, *10, *17)	
*1/*1	5 (38.5)
*1/*17	4 (30.8)
*17/*17	1 (7.7)
*1/*2	2 (15.4)
*2/*17	1 (7.7)
*CYP2C9*^‡^ (tested for *2, *3, *5, *6, *8, *11)	
*1/*1	10 (76.9)
*1/*3	2 (15.4)
*1/*6	1 (7.7)
*CYP2D6*^§^ (tested for *10, *2, *17, *41, *3, *4, *6, *9, *8, *7, *29, copy number)	
*1/*1	2 (15.4)
*1/*17	1 (7.7)
*1/*2	2 (15.4)
*1/*4	1 (7.7)
*1/*4 + duplication	1 (7.7)
*1/*5	1 (7.7)
*2/*2	1 (7.7)
*2/*4 + duplication	1 (7.7)
*2/*17	2 (15.4)
*2/*29	1 (7.7)
*CYP3A5* (tested for *3, *6, *7)	
*1/*1	2 (15.4)
*1/*3	3 (23.1)
*1/*6	2 (15.4)
*3/*3	4 (30.8)
*6/*7	2 (15.4)
*SLCO1B1*^¶^ (tested for *5)	
*1/*1	12 (92.3)
*1/*5	1 (7.7)
*CYP2C Cluster* (tested for rs12777823)	
G/G	10 (76.9)
A/G	3 (23.1)
*CYP4F2* (tested for *3)	
*1/*1	8 (61.5)
*1/*3	5 (38.5)
*VKORC1* (tested for 1639G > A)	
G/G	8 (61.5)
A/G	5 (38.5)
Values are expressed as N (%)	

^†^ Five participants were on omeprazole, 1 on esomeprazole, 1 on pantoprazole, 1 on clopidogrel and 1 on escitalopram; ^‡^ One participant was on meloxicam, 1 on ibuprofen and 1 on celecoxib; ^§^ Two participants were on oxycodone and 2 were on ondansetron; ^¶^ Five participants were on atorvastatin.

**Table 3:** Factors to Address in Patient Counseling to Promote PGx Testing Uptake.

Patients Are Motivated to Undergo Testing When They	To Attend to These Concerns/Interests:
want better medication options	the impact of medications on their body needing optimal medications for themselves
receive a clear explanation about testing	what PGx testing is the logistical feasibility of testing privacy concerns
value or have an interest in science/medicine	having scientific or medical curiosity wanting to contribute to science or research
have misconceptions about results addressed	identifying genetic predispositions—results that can be shared information with family members

**Table 4:** Patients’ Preferences for Receiving PGx Results.

Patients Want to ReceivePGx Testing Results via	For These Reasons
a sharable format	to promote better care management across providersto share results with family membersto understand and digest information on their ownto receive more detailed results
a clinical appointment(in-person, telemedicine, phone)	to explain the meaning of results to discuss the relevance of results to current medicationsto ask questions about their PGx testing results

## Data Availability

The data presented in this study are available on reasonable request from the corresponding author. The data are not publicly available due to participant privacy.
